# Clinical outcomes of radial probe endobronchial ultrasound using a guide sheath for diagnosis of peripheral lung lesions in patients with pulmonary emphysema

**DOI:** 10.1186/s12931-019-1149-0

**Published:** 2019-08-06

**Authors:** Kyu Min Lee, Geewon Lee, Ahreum Kim, Jeongha Mok, Ji Won Lee, Yeon Joo Jeong, Eun-Jung Jo, Mi Hyun Kim, Kwangha Lee, Ki Uk Kim, Hye-Kyung Park, Min Ki Lee, Jung Seop Eom

**Affiliations:** 1 Department of Internal Medicine, Pusan National University School of Medicine, 179 Gudeok-ro, Seo-gu, Busan, 602-739 Korea; 2 Department of Radiology, Pusan National University School of Medicine, Busan, Republic of Korea; 3 Biostatistics Team of Regional Center for Respiratory Diseases, Pusan National University Hospital, Busan, Republic of Korea; 4 Biomedical Research Institute, Pusan National University Hospital, Busan, Republic of Korea

**Keywords:** Bronchoscopy, Diagnosis, Lung neoplasms, Pulmonary emphysema, Ultrasound

## Abstract

**Background:**

Generally, structural destruction of lung parenchyma, such as pulmonary emphysema, is considered to be related to the low diagnostic yields and high complication rates of lung biopsies of peripheral lung lesions. Currently, little is known about the clinical outcomes of using endobronchial ultrasound with a guide sheath (EBUS-GS) to diagnose peripheral lesions in patients with emphysema.

**Methods:**

This retrospective study was performed to identify the clinical outcomes of EBUS-GS in patients with pulmonary emphysema. This study included 393 consecutive patients who received EBUS-GS between February 2017 and April 2018. The patients were classified according to the severity of their emphysema, and factors possibly contributing to a successful EBUS-GS procedure were evaluated.

**Results:**

The overall diagnostic yield of EBUS-GS in patients with no or mild emphysema was significantly higher than in those with moderate or severe pulmonary emphysema (78% vs. 61%, *P* = 0.007). There were no procedure-related complications. The presence of a bronchus sign on CT (*P* <  0.001) and a “within the lesion” status on EBUS (*P* = 0.009) were independently associated with a successful EBUS-GS procedure. Although the diagnostic yield of EBUS-GS in patients with moderate-to-severe emphysema was relatively low, a bronchus sign and “within the lesion” status on EBUS were contributing factors for a successful EBUS-GS.

**Conclusions:**

EBUS-GS is a safe procedure with an acceptable diagnostic yield, even when performed in patients with pulmonary emphysema. The presence of a bronchus sign and “within the lesion” status on EBUS were predictors of a successful procedure.

**Electronic supplementary material:**

The online version of this article (10.1186/s12931-019-1149-0) contains supplementary material, which is available to authorized users.

## Background

Low-dose computed tomography (LDCT) is widely used for lung cancer screening in high-risk individuals such as those with pulmonary fibrosis or chronic obstructive pulmonary disease, and its value for reducing mortality rate was clearly demonstrated in the US National Lung Screening Trial [1]. Although the use of LDCT to screen high-risk populations has resulted in a decrease in lung cancer mortality of 20% compared with screening using chest radiographs, high false-positive rates and the low prevalence of lung cancer are still considered major limitations to its widespread use [2, 3]. In this respect, pulmonary physicians and radiologists may find it challenging to distinguish early stage lung cancer from a benign lung nodule on LDCT [4, 5].

The National Comprehensive Cancer Network recommends histological examination for patients with a solid nodule greater than 8 mm on the initial LDCT screen [6]. Lung biopsy for a peripheral lung lesion is traditionally performed using transthoracic needle aspiration, bronchoscopy, or surgical wedge resection [5]. High-risk individuals who receive LDCT screening for lung cancer have a smoking history of more than 30 pack-years [7], and because of the possibility of reduced lung function or advanced lung destruction such as that due to pulmonary emphysema, a less invasive strategy for collecting lung tissue is required [8].

Radial probe endobronchial ultrasound using a guide sheath (EBUS-GS) has been widely used to diagnose peripheral lung lesions, with an acceptable diagnostic yield and a low complication rate [9–11]. Until now, only one previous study has shown that pulmonary emphysema may be a risk factor for pneumothorax after EBUS-GS [12]. Structural destruction of lung parenchyma, such as pulmonary emphysema, is considered to be related to the low diagnostic yields and high complication rates of lung biopsies. At present, the accuracy and safety profile of EBUS-GS in patients with pulmonary emphysema remains unclear. Thus, we used a prospectively collected database to identify the clinical outcomes of EBUS-GS in patients with pulmonary emphysema.

## Methods

### Study population

This retrospective study was performed using an EBUS-GS database to investigate the clinical outcomes of EBUS-GS in patients with pulmonary emphysema. The patients were examined between February 2017 and April 2018 at Pusan National University Hospital, a university-affiliated tertiary referral hospital in Busan, Republic of Korea. A total of 393 patients with peripheral lung lesions who received EBUS-GS during the study period were selected for the present study. The Institutional Review Board of Pusan National University Hospital approved this study, and the requirement for informed consent was waived because of the retrospective nature of the study (no. H-1809-013-071).

### CT scan and emphysema severity

CT scans were obtained in the full-inspiratory state using a commercial CT scanner (Revolution CT; GE Healthcare, Milwaukee, WI, USA). Images of the whole thorax were taken using the following CT parameters: 120 kVp; 100–250 mAs; tube rotation, 0.5; and slice thickness, 0.625 mm with an interval of 0.625 mm. Axial, coronal, and sagittal images were displayed with mediastinal (width, 400 Hounsfield units [HU]; level, 20 HU) and lung window settings (width, 1500 HU; level, − 700 HU).

A peripheral lung lesion was defined as an intrapulmonary lesion beyond the segmental bronchus visible on the axial CT scan. The mean diameter of a peripheral lung lesion was defined as the mean of the maximum transverse diameter and its perpendicular diameter on axial images with a lung window setting. As in a previous study, peripheral lung lesions were classified as solid, ground-glass opacity, mixed, or cavitary [13]. Bronchus sign on CT scan was defined as the presence of a bronchus leading directly to a peripheral lung lesion.

Thin-section CT scans were reviewed by an experienced thoracic radiologist. Emphysema was defined as a low-attenuation lung area lacking a distinct wall [14]. The extent of pulmonary emphysema was visually estimated according to a previous guideline [15], and patients were classified into three groups: mild, moderate, and severe emphysema. Briefly, mild emphysema was defined as scattered centrilobular lucencies, usually separated by large regions of normal lung, and involving an estimated 0.5–5% of a lung zone, or small (≤ 1 cm) juxtapleural lucencies. Moderate emphysema was defined as many well-defined lucencies occupying more than 5% of any lung zone. Severe emphysema included confluent centrilobular emphysema, advanced destructive emphysema, and substantial paraseptal emphysema [15].

### EBUS-GS procedure

**Fig. 1:**
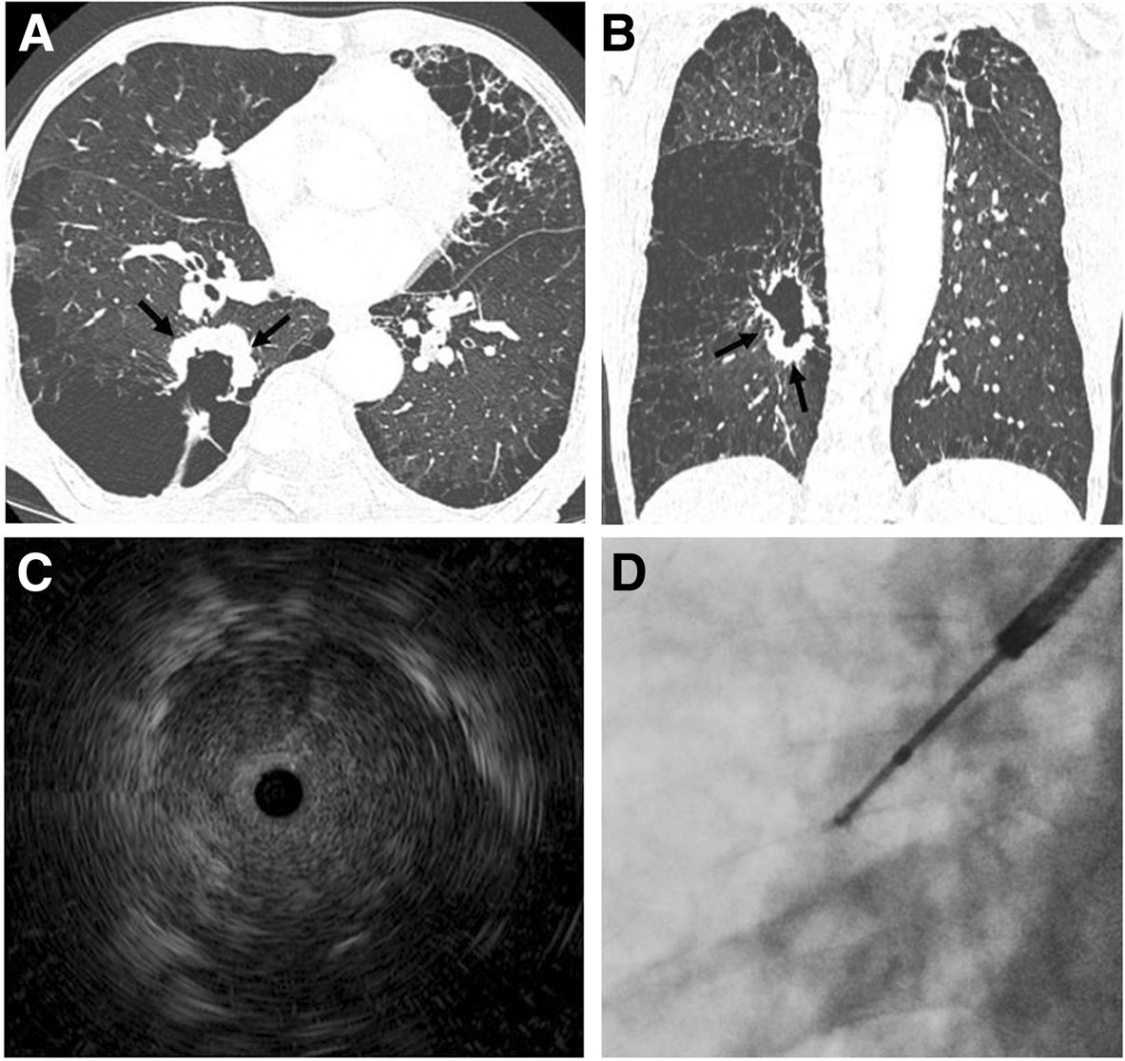
Representative case of EBUS-GS in a patient with severe pulmonary emphysema. **a** and **b** A 31 × 37 mm cavitary lesion in the right lower lobe is shown on axial and coronal computed tomography scans of a patient with severe emphysema. Because of the advanced lung destruction around the tumor (black arrow), it was impossible to perform percutaneous needle aspiration for a histological examination. **c** Radial probe EBUS shows “within the lesion” status. **d** A transbronchial lung biopsy was performed under fluoroscopic guidance, and squamous cell carcinoma was diagnosed. EBUS-GS, endobronchial ultrasound using a guide sheath

**Fig. 2:**
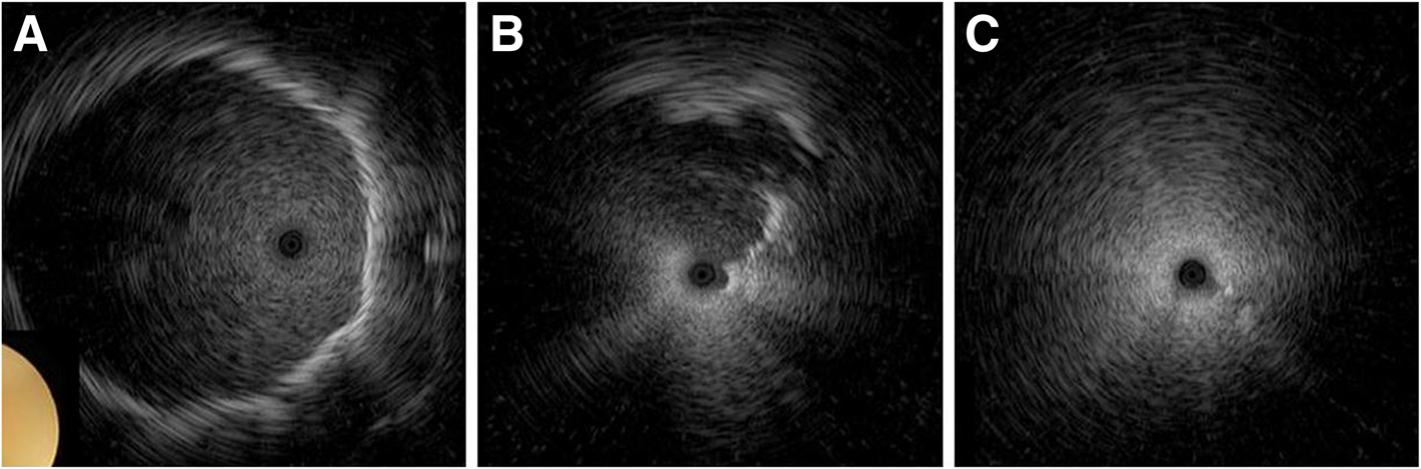
EBUS image according to the relationship between the peripheral lung lesion and the bronchus. The radial EBUS was positioned inside the peripheral lung lesion (**a**, within), in the bronchus adjacent to the lung lesion (**b**, adjacent to) and outside the lung lesion (**c**, outside). EBUS, endobronchial ultrasound

EBUS-GS was performed using the standard techniques of Kurimoto [9], and all procedures during the study period were performed without any assistance from novel navigation modalities such as electromagnetic navigation bronchoscopy or virtual bronchoscopy [10, 11]. A representative case of EBUS-GS in a patient with pulmonary emphysema is shown in Fig. 1. Briefly, using a thin-section chest CT scan for guidance, a thin bronchoscope (BF-P260F; Olympus, Tokyo, Japan) was advanced as close as possible to the target peripheral lesion under conscious sedation. Then, a 20 MHz radial EBUS probe (UM-S20-17S; Olympus), covered with a GS (K-201; Olympus) was introduced through the working channel of the bronchoscope to precisely locate the target lung lesion. Following previously reported classifications [9, 16, 17], radial probe EBUS findings of the target peripheral lesion were classified as within, adjacent to, or outside of the lesion (Fig. 2). After identifying the target lesion on the radial probe EBUS, subsequent brush cytology and forceps biopsy were performed under X-ray fluoroscopic guidance. To prevent infectious complications, prophylactic antibiotics were empirically prescribed for patients with cavitary lesions or structurally damaged lung, according to previous reports [18, 19].

### Complications related to EBUS-GS

Any complications that developed after EBUS-GS were evaluated as described in previous studies [18, 19]. Briefly, severe hemorrhage was defined as procedure-related bleeding requiring endotracheal intubation, transfusion, or another invasive procedure for hemostasis. Chest radiographs were performed 4 h after the procedure and on the following day to check the iatrogenic pneumothorax after EBUS-GS. Infectious complications, air embolisms, respiratory failure, and premature termination of the procedure due to an unexpected complication during EBUS-GS were recorded by the physicians performing the procedures.

### Statistical analysis

All results are presented as median values (interquartile range [IQR]) for continuous variables and as numbers (percentages) for categorical variables, as appropriate. The categorical data were compared using Pearson’s chi-square or Fisher’s exact test, and the Mann–Whitney *U* test was used to compare continuous variables. Multivariate logistic regression analysis was used to examine independent factors related to successful EBUS-GS. A *P-*value < 0.1 was considered significant in the univariate analyses, and *P*-values < 0.05 were considered significant in all other analyses. SPSS for Windows (ver. 22.0 SPSS Inc., Chicago, IL, USA) was used for the statistical analyses.

## Results

### Study population

Of the 393 study patients who received EBUS-GS, pulmonary emphysema was found in 129 patients (33%). The baseline characteristics of the patients with and without pulmonary emphysema are shown in Table 1. The proportion of male gender was higher in study subjects with pulmonary emphysema than in those without emphysema on CT (98% vs. 53%, *P* <  0.001). The median forced expiratory volume in 1 s and the forced expiratory volume in 1 s/forced vital capacity ratio were significantly lower in patients with pulmonary emphysema than in those without pulmonary emphysema (79% predicted value vs. 86% predicted value, *P* <  0.001 for forced expiratory volume in 1 s; 69% vs. 75%, *P* <  0.001 for forced vital capacity). Otherwise, there were no statistically significant differences in baseline characteristics between patients with and without pulmonary emphysema. The clinical diagnoses of all study patients are presented in Table 2. Of the 129 patients with pulmonary emphysema, mild, moderate, and severe emphysema was found on axial CT in 70 (54%), 45 (35%), and 14 (11%) patients, respectively.

**Table 1:** Baseline characteristics of the study patients

Variables	With emphysema (*n* = 129)	Without emphysema (*n* = 264)	*P*-value
Age, years	71 (65–76)	69 (61–75)	0.064
Male gender	126 (98)	139 (53)	< 0.001
Mean diameter of lesion, mm	28 (21–39)	27 (19–35)	0.135
Distance from pleura, mm	1 (0–25)	10 (0–23)	0.289
Pulmonary function test^a^			
FEV_1_, % predicted value	79 (66–91)	86 (75–97)	< 0.001
FVC, % predicted value	82 (74–90)	85 (75–96)	0.066
FEV_1_/FVC, %	69 (60–75)	75 (70–80)	< 0.001
Lesion location			
Right upper lobe	36 (28)	64 (24)	0.525
Right middle lobe	12 (9)	19 (7)	
Right lower lobe	31 (24)	64 (24)	
Left upper division	22 (17)	63 (24)	
Left lingular division	7 (5)	8 (3)	
Left lower lobe	21 (16)	46 (17)	
Character of lesion on CT scan			
Solid	113 (88)	234 (89)	0.456
Mixed	5 (4)	16 (6)	
Ground-glass opacity	2 (2)	4 (2)	
Cavitary	9 (7)	10 (4)	

^a^ Pulmonary function test results were available for 245 patients without pulmonary emphysema (93%)

*IQR* interquartile range, *FEV*_*1*_ forced expiratory volume in 1 s, *FVC* forced vital capacity, *CT* computed tomography

**Table 2:** Clinical diagnoses of all the patients who underwent EBUS-GS

Variables	No. (%)
Patients with pulmonary emphysema	
Diagnosed by EBUS-GS (*n* = 91)	
Lung cancer	85 (93)
Pulmonary tuberculosis	4 (4)
NTM lung disease	1 (1)
Organizing pneumonia	1 (1)
Undiagnosed on EBUS-GS (*n* = 38)	
Lung cancer	19 (50)
Pneumonia	3 (8)
Pulmonary tuberculosis	1 (3)
Lymphoma	1 (3)
Unknown	14 (37)
Patients without pulmonary emphysema	
Diagnosed by EBUS-GS (*n* = 204)	
Lung cancer	187 (91)
Pulmonary tuberculosis	7 (3)
Pneumonia or lung abscess	3 (2)
Metastatic lung nodules	3 (2)
Sarcoidosis	1 (1)
Fungal infection	1 (1)
NTM lung disease	1 (1)
Lymphoma	1 (1)
Undiagnosed on EBUS-GS (*n* = 60)	
Lung cancer	23 (38)
Metastatic lung nodules	3 (5)
Fungal infection	2 (3)
Pneumonia or lung abscess	2 (3)
Pulmonary tuberculosis	1 (2)
NTM lung disease	1 (2)
Interstitial lung disease	1 (2)
Unknown	27 (45)

*EBUS-GS* endobronchial ultrasound using a guide sheath, *NTM* nontuberculous mycobacteria

### Diagnostic yield

**Fig. 3:**
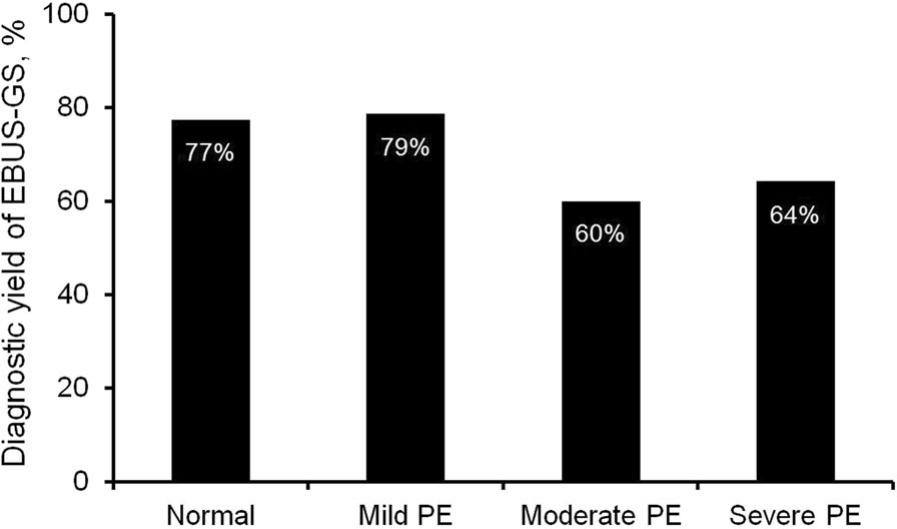
Diagnostic yield of EBUS-GS according to the severity of emphysema on CT scan. EBUS-GS, endobronchial ultrasound using a guide sheath; PE, pulmonary emphysema

The diagnostic yields of EBUS-GS in patients with and without pulmonary emphysema were 71 and 77%, respectively, and were not significantly different (*P* = 0.148). In addition, there was no statistically significant difference in the diagnostic yield of EBUS-GS between patients with mild, moderate, or severe pulmonary emphysema (79, 60, and 64% for mild, moderate, and severe pulmonary emphysema, respectively, *P* = 0.089). However, the overall diagnostic yield of EBUS-GS in patients with no or mild emphysema was significantly higher than in those with moderate or severe pulmonary emphysema (78% for no or mild pulmonary emphysema vs. 61% for moderate or severe pulmonary emphysema, *P* = 0.007; Fig. 3).

### Factors associated with successful EBUS-GS in patients with emphysema

Factors affecting the diagnostic yield of EBUS-GS in patients with pulmonary emphysema are compared in Table 3. Univariate analysis revealed that, in comparison with those who did not have a successful EBUS-GS, those patients who had a successful EBUS-GS were more likely to have a lung lesion with a large mean diameter (29 mm vs. 23 mm, *P* = 0.008), a greater proportion of mild pulmonary emphysema (60% vs. 40%, *P* = 0.029), the presence of a bronchus sign on CT scan (96% vs. 40%, *P* <  0.001), and “within the lesion” status on radial probe EBUS (96% vs. 50%, *P* <  0.001). Multivariate logistic regression analysis, which was performed to verify the independent factors associated with successful EBUS-GS, showed that the presence of a positive bronchus sign (odds ratio, 33.426; 95% confidence interval, 7.550–147.993; *P* <  0.001) and “within the lesion” status on radial probe EBUS (odds ratio, 7.641; 95% confidence interval, 1.662–35.129; *P* = 0.009) were independently associated with a successful EBUS-GS in patients with pulmonary emphysema (Table 4).

**Table 3:** Factors possibly affecting the diagnostic yield of EBUS-GS in patients with pulmonary emphysema

Variables	Success (*n* = 91)	Failure (*n* = 38)	*P*-value
Age, years	72 (64–76)	70 (65–75)	0.351
Male gender	88 (97)	38 (100)	0.555
Mean diameter of the lung lesion, mm	29 (23–39)	23 (17–37)	0.008
Severity of pulmonary emphysema			
Mild	55 (60)	15 (40)	0.029
Moderate or severe	36 (40)	23 (60)	
Distance from pleura to lung lesion, mm	8 (0–27)	0 (0–23)	0.231
Pulmonary function test			
FEV_1_, % predicted value	80 (64–89)	77 (70–92)	0.953
FVC, % predicted value	81 (74–89)	84 (73–91)	0.468
FEV_1_/FVC, %	69 (60–75)	67 (60–75)	0.614
Lesion location			
Right upper lobe	27 (30)	9 (24)	0.570
Right middle lobe	9 (10)	3 (8)	
Right lower lobe	23 (25)	8 (21)	
Left upper division	16 (18)	6 (16)	
Left lingular division	3 (3)	4 (11)	
Left lower lobe	13 (14)	8 (21)	
Bronchus sign			
Positive	87 (96)	15 (40)	< 0.001
Negative	4 (4)	23 (60)	
Character of lesion on CT			
Solid	81 (89)	35 (92)	1.000
Mixed	1 (1)	0 (0)	
Ground-glass opacity	1 (1)	0 (0)	
Cavitary	8 (9)	3 (8)	
EBUS finding			
Within lesion	87 (96)	19 (50)	< 0.001
Adjacent to or outside lesion	4 (4)	19 (50)	

*EBUS-GS* endobronchial ultrasound using a guide sheath, *FEV*_*1*_ forced expiratory volume in 1 s, *FVC* forced vital capacity, *CT* computed tomography

**Table 4:** Multivariate logistic regression analysis to identify independent factors associated with successful EBUS-GS

	Odds ratio (95% confidence interval)	*P*-value
Age (per year)	0.958 (0.900–1.020)	0.181
Mean diameter of lung lesion (per mm)	0.993 (0.956–1.031)	0.712
Mild pulmonary emphysema	1.001 (0.328–3.053)	0.998
Positive bronchus sign	33.426 (7.550–147.993)	< 0.001
“Within the lesion” EBUS finding	7.641 (1.662–35.129)	0.009

*EBUS-GS* endobronchial ultrasound using a guide sheath

Table 5 shows how the lesion characteristics were related to the severity of pulmonary emphysema. A bronchus sign was significantly more frequent in patients with mild pulmonary emphysema than in those with moderate-to-severe pulmonary emphysema (89% vs. 68%, *P* = 0.004), and patients with mild pulmonary emphysema were significantly more likely to show “within the lesion” status on radial probe EBUS than those with moderate-to-severe pulmonary emphysema (90% vs. 73%, *P* = 0.011). Otherwise, there were no significant differences between the two groups, including the mean lesion diameter, the distance from the pleura to the lung lesion, and the characteristics of the lesion on CT. An additional multivariate logistic regression analysis was conducted to identify factors predicting successful EBUS-GS in patients with moderate-to-severe pulmonary emphysema (See Additional file 1: Table S1). The bronchus sign on CT scan (odds ratio, 23.459; 95% CI, 3.464–158.868; *P* = 0.001) and “within the lesion” status on radial probe EBUS (odds ratio, 10.512; 95% CI, 1.363–81.047; *P* = 0.024) were independently associated with a successful EBUS-GS in patients with moderate-to-severe pulmonary emphysema.

**Table 5:** Comparisons of EBUS-GS and other characteristics according to the severity of pulmonary emphysema

Variables	Mild emphysema (*n* = 70)	Moderate-to-severe emphysema (*n* = 59)	*P*-value
Mean diameter of lesion, mm	27 (22–39)	28 (19–39)	0.709
Distance from pleura to lung lesion, mm	3 (0–22)	0 (0–27)	0.927
Number of brushing cytology tests	3 (3–3)	3 (2–3)	0.371
Number of biopsies	6 (6–6)	6 (6–7)	0.379
Lesion location			
Right upper lobe	25 (36)	11 (19)	0.072
Right middle lobe	4 (6)	8 (14)	
Right lower lobe	16 (23)	15 (25)	
Left upper division	12 (17)	10 (17)	
Left lingular division	1 (1)	6 (10)	
Left lower lobe	12 (17)	9 (15)	
Bronchus sign			
Positive	62 (89)	40 (68)	0.004
Negative	8 (11)	19 (32)	
Character of lesion on CT			
Solid	62 (89)	54 (92)	0.484
Mixed	0 (0)	1 (2)	
Ground-glass opacity	1 (1)	0 (0)	
Cavitary	7 (10)	4 (7)	
EBUS finding			
Within lesion	63 (90)	43 (73)	0.011
Adjacent to or outside lesion	7 (10)	16 (27)	

*FEV*_*1*_ forced expiratory volume in 1 s, *FVC* forced vital capacity, *EBUS* endobronchial ultrasound, *CT* computed tomography

### Complications

No mortalities or life-threatening complications were associated with the procedure during the study period. None of the patients with pulmonary emphysema developed a pneumothorax, severe hemorrhage, air embolism, or pulmonary infection.

## Discussion

In general, the incidence rate of pneumothorax of any type after percutaneous lung biopsy is 15% [5], and the presence of pulmonary emphysema is particularly closely associated with pneumothorax after percutaneous lung biopsy [20]. The destruction of lung parenchyma and poor lung function in patients with pulmonary emphysema renders percutaneous lung biopsy difficult. By contrast, guided bronchoscopy such as EBUS-GS has a low complication rate with an acceptable diagnostic yield [18, 21, 22]. A previous study reported that the overall complication rate, including iatrogenic pneumothorax, is 1.3% [18]; however, we are not aware of any previous study investigating the diagnostic yield or safety profile of EBUS-GS in patients with pulmonary emphysema. In the present study, the overall diagnostic yield of EBUS-GS for peripheral lung lesions in the 129 patients with pulmonary emphysema was 71%, without any complications occurring. Many physicians hesitate to make pathologic diagnoses of peripheral lung lesions in patients with pulmonary emphysema because of its associated complications and low diagnostic yields; however, our results suggest that a pathological diagnosis of a peripheral lung lesion can be made safely using EBUS-GS.

Previous studies have reported that the position of the radial EBUS probe during the procedure, lesion size, malignant status, and the bronchus sign on CT contribute to successful EBUS-GS [9, 16, 23, 24]. Similarly, in the present study, a positive bronchus sign on CT and “within the lesion” status on radial probe EBUS were significantly associated with successful EBUS-GS in patients with pulmonary emphysema. Both the EBUS findings and bronchus sign on CT represent the relationship between the target lung lesion and the peripheral bronchus [25]. Our results suggest that the relationship between the peripheral lesion and the bronchus is an important factor, even if EBUS-GS is performed in patients with pulmonary emphysema.

The diagnostic yield of EBUS-GS in patients with mild pulmonary emphysema was similar to that in those without emphysema. Moreover, we found that the diagnostic yield in patients with moderate-to-severe emphysema was significantly lower than that in patients with no or mild pulmonary emphysema. Emphysema is characterized by the destruction of lung parenchyma and alveolar attachment [26]. In general, detection of the bronchus sign in a patient with advanced pulmonary emphysema is difficult because of the destruction of the lung parenchyma. Even if a reconstructed thin-section CT is prepared for EBUS-GS, it is often impossible to differentiate an emphysematous change from the peripheral bronchus on CT. Moreover, narrow and obliterated small airway lumens in patients with moderate-to-severe pulmonary emphysema may contribute to the clinical outcomes of EBUS-GS [27]. However, our results indicate that a clear indication of the bronchus sign on CT scan and “within the lesion” status on EBUS are related to a successful diagnosis of EBUS-GS. Although the diagnostic yield of EBUS-GS in patients with moderate-to-severe pulmonary emphysema was relatively low, the careful selection of patients according to the bronchus sign on CT could lead to a high rate of successful procedures.

EBUS-GS is generally considered to be a safe procedure in comparison with surgical biopsy and percutaneous lung biopsy [5]. Hayama et al. reported an overall complication rate of 1.3% for EBUS-GS performed on 965 peripheral lung lesions (pneumothorax, 0.8%; pulmonary infection, 0.5%) [18], while Huang et al. reported that the incidence of pneumothorax after EBUS-GS was 3.3% in 399 patients with peripheral lung lesions [12]. However, there were no procedure-related EBUS-GS complications in the present study. In the entire study population with pulmonary emphysema, fluoroscopy was used for the forceps biopsy and brushing cytology through a guide sheath, after precise localization of the peripheral lung lesion with the radial probe EBUS. During EBUS-GS, fluoroscopy helps prevent the forceps or brush from moving out of the exact target location in the peripheral lung lesion. Our results suggest that fluoroscopic guidance could help avoid an iatrogenic pneumothorax, even if EBUS-GS is performed in patients with pulmonary emphysema. Moreover, in the present study, prophylactic antibiotics were prescribed to prevent pulmonary infection in high-risk patients, such as those with a cavitary lesion or a structurally damaged lung. Our results suggest that prophylactic antibiotics in high-risk patients can help prevent infectious complications of EBUS-GS, especially in patients with pulmonary emphysema.

Some limitations of this study should be acknowledged. First, this retrospective study was conducted at a single institution, and there is the potential that selection bias could have influenced our results; therefore, it is difficult to generalize the findings. Second, the number of enrolled patients was relatively small. In particular, the EBUS-GS complication rate may have been underestimated because of the small study population. Third, all EBUS-GSprocedures were performed without the assistance of a navigation system, such as electromagnetic navigation bronchoscopy or virtual bronchoscopy [10, 11]. To verify our results, a prospective study including a large number of patients with pulmonary emphysema is needed.

## Conclusions

EBUS-GS was shown to be a safe procedure with an acceptable diagnostic yield, even when performed in patients with pulmonary emphysema. The presence of a bronchus sign and “within the lesion” status on radial probe EBUS were useful predictors for a successful diagnosis.

## Additional file

Additional file 1: **Table S1.** The affecting factors for diagnostic yield of EBUS-GS in moderate-to-severe pulmonary emphysema. (DOCX 17 kb)

## Data Availability

Please contact author for data requests.

## Abbreviations

CT: Computed tomography
EBUS: Endobronchial ultrasound
EBUS-GS: Endobronchial ultrasound using a guide sheath
HU: Hounsfield units
IQR: Interquartile range
LDCT: Low dose computed tomography
NTM: Nontuberculous mycobacteria
