# Supplementary material for: Clinical outcomes of radial probe endobronchial ultrasound using a guide sheath for diagnosis of peripheral lung lesions in patients with pulmonary emphysema
Source: Respir Res. 2019 Aug 6;20:177. doi: 10.1186/s12931-019-1149-0 (PMC6683511; doi:10.1186/s12931-019-1149-0)
Supplement: Supplementary file 1 — Table S1. The affecting factors for diagnostic yield of EBUS-GS in moderate-to-severe pulmonary emphysema. (DOCX 17 kb) [file 12931_2019_1149_MOESM1_ESM.docx]

Additional file 1: Table S1. The affecting factors for diagnostic yield of EBUS-GS in moderate-to-severe pulmonary emphysema.

| Variables | Success (n =36) | Failure (n =23) | *P*-value |
| --- | --- | --- | --- |
| Age, years | 70 (64–76) | 71 (66–76) | 0.446 |
| Male gender | 36 (100) | 23 (100) |  |
| Mean diameter of the lung lesion, mm | 34 (24–41) | 25 (17–33) | 0.018 |
| Distance from pleura to lung lesion, mm | 14 (0–26) | 14 (0–33) | 0.576 |
| Pulmonary function test |  |  |  |
| FEV_1_, % predicted value | 75 (63–89) | 75 (55–91) | 1.000 |
| FVC, % predicted value | 83 (73–90) | 84 (73–93) | 0.619 |
| FEV_1_/FVC, % | 64 (54–73) | 62 (54–70) | 0.597 |
| Lesion location |  |  | 0.497 |
| Right upper lobe | 5 (14) | 6 (26) |  |
| Right middle lobe | 6 (17) | 2 (9) |  |
| Right lower lobe | 10 (28) | 5 (22) |  |
| Left upper division | 7 (19) | 3 (13) |  |
| Left lingular division | 2 (6) | 4 (17) |  |
| Left lower lobe | 6 (17) | 3 (13) |  |
| Bronchus sign |  |  | < 0.001 |
| Positive | 34 (94) | 6 (26) |  |
| Negative | 2 (6) | 17 (74) |  |
| Character of lesion on CT scan |  |  | 0.595 |
| Solid | 32 (89) | 22 (96) |  |
| Mixed | 1 (3) | 0 (0) |  |
| Ground glass opacity | 0 (0) | 0 (0) |  |
| Cavitary | 3 (8) | 1 (4) |  |
| EBUS finding |  |  | < 0.001 |
| Within the lesion | 34 (94) | 9 (39) |  |
| Adjacent to or outside | 2 (6) | 14 (61) |  |

EBUS-GS, endobronchial ultrasound using a guide sheath; FEV_1_, forced expiratory volume in one second; FVC, forced vital capacity.
